# *GmARP* is Related to the Type III Effector NopAA to Promote Nodulation in Soybean (*Glycine max*)

**DOI:** 10.3389/fgene.2022.889795

**Published:** 2022-05-27

**Authors:** Jinhui Wang, Chao Ma, Shengnan Ma, Haiyang Zheng, Haojie Feng, Yue Wang, Jiangxu Wang, Chunyan Liu, Dawei Xin, Qingshan Chen, Mingliang Yang

**Affiliations:** ^1^ College of Agriculture, Key Laboratory of Soybean Biology in Chinese Ministry of Education, Northeast Agricultural University, Harbin, China; ^2^ Heilongjiang Academy of Agricultural Sciences, Harbin, China

**Keywords:** soybean, symbiosis, nodulation, glycosyl hydrolase, QTL

## Abstract

Type III effectors secreted by rhizobia regulate nodulation in the host plant and are important modulators of symbiosis between rhizobia and soybean (*Glycine max*), although the underlying mechanisms are poorly understood. Here, we studied the type III effector NopAA in *Sinorhizobium fredii* HH103, confirming its secretion into the extracellular environment under the action of genistein. The enzyme activity of NopAA was investigated *in vitro*, using xyloglucan and β-glucan as substrates. NopAA functions were investigated by the generation of a NopAA mutant and the effects of NopAA deficiency on symbiosis were analyzed. Soybean genes associated with NopAA were identified in a recombinant inbred line (RIL) population and their functions were verified. NopAA was confirmed to be a type III effector with glycosyl hydrolase activity, and its mutant did not promote nodulation. Quantitative trait locus (QTL) analysis identified 10 QTLs with one, *Glyma.19g074200* (*GmARP*), found to be associated with NopAA and to positively regulate the establishment of symbiosis*.* All these results support the hypothesis that type III effectors interact with host proteins to regulate the establishment of symbiosis and suggest the possibility of manipulating the symbiotic soybean–rhizobia interaction to promote efficient nitrogen fixation.

## Introduction

Soybean is an important food crop, could provide rich plant protein and oil for human. With the increasing demand for protein and oil, more and more attention has been paid to soybean agricultural production (Li et al., 2019). In agricultural production, the yield of soybean is increased by applying a large amount of industrial nitrogen fertilizer (Wang et al., 2020a). However, excess application of nitrogen fertilizers has had adverse effects on the environment, leading also to economic and health concerns (Jordan et al., 2011; Chen et al., 2017; Guo and Wang, 2021). The use of symbiotic nodule-forming soybean maintains soil fertility and sustainable crop production, leading to reduced requirements for chemical N fertilizers in agricultural systems (Goyal et al., 2021). Many native *Rhizobium* species have been isolated and inoculated into soybean agricultural production to reduce the amount of applied N fertilizer (Chen et al., 2021). Rhizobia inoculations benefit the production of soybean as they promote plant growth by producing hormones, antibiotics, vitamins, and siderophores (Roy et al., 2020); in addition, rhizobia can enhance disease resistance (Plett et al., 2021; Zboralski et al., 2022).

The establishment of symbiosis system is a complex process, which requires signal exchange between soybean and rhizobia (Roy et al., 2020). In rhizobia, six secretion systems, namely, type I to VI systems, have been identified and classified according to their function (Freiberg et al., 1997; Krehenbrink and Downie, 2008; Pukatzki et al., 2009; Tampakaki, 2014; van Ulsen et al., 2014). Each secretion system plays a different role in symbiotic nitrogen fixation, with the type III secretion system (T3SS) being the most widely studied (Kambara et al., 2009; Miwa and Okazaki, 2017). The T3SS secretes nodulation outer proteins (Nops) into host cells which play key roles in the establishment of symbiosis. Several Nops have been identified in *Sinorhizobium fredii* HH103, including NopD, NopL, NopM, NopP, and NopT (Ausmees et al., 2004; Bartsev et al., 2004; Dai et al., 2008). NopD and NopM have been identified by LC-MALDI and LC-ESI (Rodrigues et al., 2007), and protein domain prediction has shown that the C-terminal region of NopD contains a domain homologous to the ubiquitin-like protease Ulp1, thus suggesting that NopD may combine with SUMO (small ubiquitin-like modifier)-conjugated proteins, leading to the removal of the SUMO conjugate in HH103-infected soybean (Xiang et al., 2020; Ratu et al., 2021). NopM is an E3 ubiquitin ligase and may target the MAPK pathway in host plants during nodule formation (Xin et al., 2012). NopL and NopP are substrates for soybean kinases (Skorpil et al., 2005; Zhang et al., 2011a; Ge et al., 2016). The NopL mutant was found to inhibit nodule formation, while the NopP mutant increased nodule numbers (Wang et al., 2018; Zhang et al., 2018; Zhang et al., 2021a). These reports indicate that the various type III effectors have different roles in symbiosis. The analysis and identification of type III effector proteins in soybean could provide a good basis for understanding the functions of these proteins in symbiosis. The release of the soybean (*Glycine max*) reference genome (Lam et al., 2010; Schmutz et al., 2010) has allowed the identification of many novel genes based on quantitative trait loci (QTLs) (Qi et al., 2014a; Lu et al., 2017). The Rj2 protein was cloned and found to recognize T3SS proteins (Yang et al., 2010). *GmNNL1*, an *R* gene that interacts with the rhizobial effector NopP, was identified by GWAS (Zhang et al., 2021a). NopD promotes HH103 infection of soybean by direct or indirect regulation of *GmPP2C*, and different haplotypes of *GmPP2C* were found to vary their expression patterns in response to NopD (Wang et al., 2020b). *GmRPK* was found to be related to NopL and to promote HH103 infection (Zhang et al., 2018). Although many proteins related to type III effectors have been identified in soybean, much work is still needed to uncover the mechanisms by which these proteins function.

In this study, we aimed to identify the genes associated with NopAA in regulating nodulation. We identified NopAA as a glycosyl hydrolase that hydrolyzed xyloglucan and β-glucan into glucose. Nodule tests in 13 soybean natural varieties were used to study the effects of NopAA mutation, indicating that NopAA positively influenced symbiosis. We identified 10 QTLs related to NopAA using the Dongnong594 and Charleston germplasms. Further haplotypic, genetic, and transgenic analyses showed that *Glyma.19g074200* (*GmARP*) responded to NopAA to regulate nodulation. These findings thus provide useful information on the role of NopAA in nodulation, and the identification and study of soybean host genes can provide support for the effective utilization of symbiotic nitrogen fixation in soybean.

## Materials and Methods

### Strains, Vectors, and Primers

*S. fredii* HH103 and *Escherichia coli* DH5α and BL21 (DE3.1) were used in this study (Supplementary Table S2). HH103 was cultured in or on TY medium supplemented with rifampicin at 28°C, and all *E. coli* strains were cultured in or on LB media supplemented with appropriate antibiotics at 37°C. The plasmids used for the construction of mutants and the primers used for PCT and qRT-PCR are listed in Supplementary Table S1. Antibiotics were used at the following concentrations: 50 μg/ml rifampicin; 50 μg/ml kanamycin; 34 μg/ml chloramphenicol; 50 μg/ml spectinomycin; 50 μg/ml streptomycin.

### Construction of the NopAA Mutant

The NopAA mutant was constructed by triparental mating as previously described (Wang et al., 2019) with the kanamycin-resistant DNA fragment inserted into the coding sequence of NopAA by homologous recombination. The mutant was confirmed by PCR and Southern blotting (Chen and Kuo, 1993).

### qRT-PCR and Analysis of Nodulation Outer Proteins

HH103, the NopAA mutant, and the TtsI mutant were grown in YM medium (Yeast Mannitol Broth) in the presence or absence of genistein, and extracellular proteins were extracted as previously described (Jim é nez-Guerrero et al., 2015). Extracted RNA was subjected to qRT-PCR analysis by a Roche LightCycler LC480 (Roche, Switzerland), and the relative expression levels of *NopAA* were normalized using *16S rRNA*. All sample collections were analyzed using three independent biological replicates as well as three technical replicates.

### Enzyme Activity Assays

The NopAA genes were then independently subcloned into pET28b as *Bam*H I-*Sal* I fragments to create pET28b-NopAA. This vector was used for active protein purifications as described (Xin et al., 2012). The His-NopAA protein was purified from BL21 (DE3) *E. coli* after induction with 0.1 mM isopropylthio-β-galactoside using Ni-NTA resin beads (Qiagen, Hilden, Germany). The purified protein was identified by western blotting with an anti-His antibody and its enzyme activity was measured using pure xyloglucan and beta-glucan (Megazyme, Bray, Ireland) as substrates. Activity assays were performed by glucose as the standard and a iMark Microplate Absorbance Reader (Bio-Rad, United States) as previously described (Grishutin et al., 2004).

### Agrobacterium Infiltration Assays

*Agrobacterium*-mediated transformation of tobacco leaves was performed as described (Wang et al., 2011). *A. tumefaciens* EHA105 containing pSoy1*-NopAA*, the empty vector pSoy1, and pSoy1 *35S-NopM* were transformed into leaves of 30-day-old *Nicotiana benthamiana*. The OD_600_ of the *A. tumefaciens* culture was adjusted to 0.2 with buffer (Ma et al., 2015). Infiltrated leaves were harvested each day for analysis of electrical conductivity by a conductivity meter (Lei-ci, China) (Wang et al., 2020a).

### Nodulation Tests

The RILs population were planted in Xiangyang Farm, Harbin, Heilongjiang Province (Harbin, latitude 45°450″N, longitude 126°380″E) in 2016, and nodulation tests were performed in 2017. 100 soybean germplasms used in the study came from different ecoregions were planted in Xiangyang Farm in 2019, and nodulation tests were performed in 2020. Sterilized soybean seeds (15 seeds from each variety) were grown under greenhouse conditions in sterilized vermiculite containing a low-nitrogen nutrient solution (Zhang et al., 2018). All plants were grown in a greenhouse at 25°C with a photoperiod of 16 h. At the Vc stage (unifoliolate leaves expand), all the plants were inoculated with HH103 and the NopAA mutant. Nodulation in the germplasms was evaluated by nodule number (NN) and nodule dry weight (NDW) (28 days post-inoculation, dpi). Three different biological replicates and 15 plants for each replicate were conducted and the *t*-test was used to detect the statistical significance of differences in NN and NDW.

### Observation of Rhizobial Infection Events

DN594 and Charleston were inoculated with GFP-conjugated HH103 and NopAA mutant strains. One-centimeter samples of infected lateral roots were collected at 1 dpi, comprising ten lateral roots per plant, and infection in the entire plant was evaluated using confocal fluorescence microscopy (Zeiss LSM700, Germany). The number of infection events was then multiplied by the length of the roots. Infection events included foci, infection threads in epidermal cells (ITs), and infection threads extending into the cortical cells (rITs) (Liu et al., 2020). Ten independent lateral root segments from each plant were used for each biological replicate and three biological replicates were used for each condition.

### Quantitative Trait Locus Mapping and Identification of Genes in Quantitative Trait Locus Regions

Charleston and DN594 were used to construct the RIL population, using a genetic map that had been constructed in a previous study (Qi et al., 2014b). We used WinQTL Cartographer and composite interval mapping methods to map QTLs related to nodulation in the RIL population. QTLs for NN and NDW were defined by LOD scores greater than 3.0 (Brensha et al., 2012; Wang et al., 2020b). The reference genome of Williams 82 was used to identify genes within the QTL regions, and candidate genes were annotated according to the gene annotation information of Williams 82 (Xin et al., 2016).

### Analysis of the Expression Patterns of Candidate Genes Related to NopAA

qRT-PCR was performed to analyze the expression patterns of candidate genes in Charleston and DN594 inoculated with HH103, the NopAA mutant, and the control. Roots were harvested at several time points and rapidly submerged in liquid nitrogen, after which the total RNA was extracted by the TRIzol method. cDNA was synthesized using the PrimeScript™ RT Reagent Kit (Takara Biotech Co., Beijing, China) and qRT-PCR was performed using TB Green^®^ Premix Ex Taq™ II (Takara Biotech Co.). Relative expression levels of each gene were normalized using *GmUNK1* (*Glyma.12g020500*) (Wang et al., 2020a).

### Hairy-Root Transformation of Soybean

The *A. rhizogenes* strain K599 containing pSoy10*-GmARP-GFP*, pSoy10*-GFP*, pB7GWIWG2-*GmARP-DsRed,* and pB7GWIWG2*-DsRed* were used for hairy-root transformation as described previously (Kereszt et al., 2007). GFP and DsRED were used as markers to detect positive hairy roots using LUYOR-3415RG. Hairy roots were inoculated with HH103, the NopAA mutant, and the control to identify the influence of gene overexpression and interference on nodulation. All nodules were harvested at 28 dpi and transcript levels of *GmARP* were measured using qRT-PCR. Three different biological replicates and 15 plants for each replicate were conducted and the *t*-test was used to detect the statistical significance of differences in NN and NDW.

### Haplotype Analysis

Haplotype analysis of *GmARP* was performed in 100 soybean natural varieties. The genomic sequence of *GmARP,* including the coding sequence and the 3000-bp promoter sequence, was obtained from the resequencing of the genomes of the 100 varieties, and the genomic sequences were subjected to local BLAST analysis to obtain SNP information (Wang et al., 2020a). Haploview 4.2 software (Cambridge, MA, United States) was used for analysis using the Haps Format module, and GraphPad Prism 8 software was used to analyze the correlations between the *GmARP* haplotypes and nodule traits in the 100 natural varieties (Li et al., 2019).

## Results

### NopAA is Induced by Genistein and Secreted by Rhizobia

**FIGURE 1:**
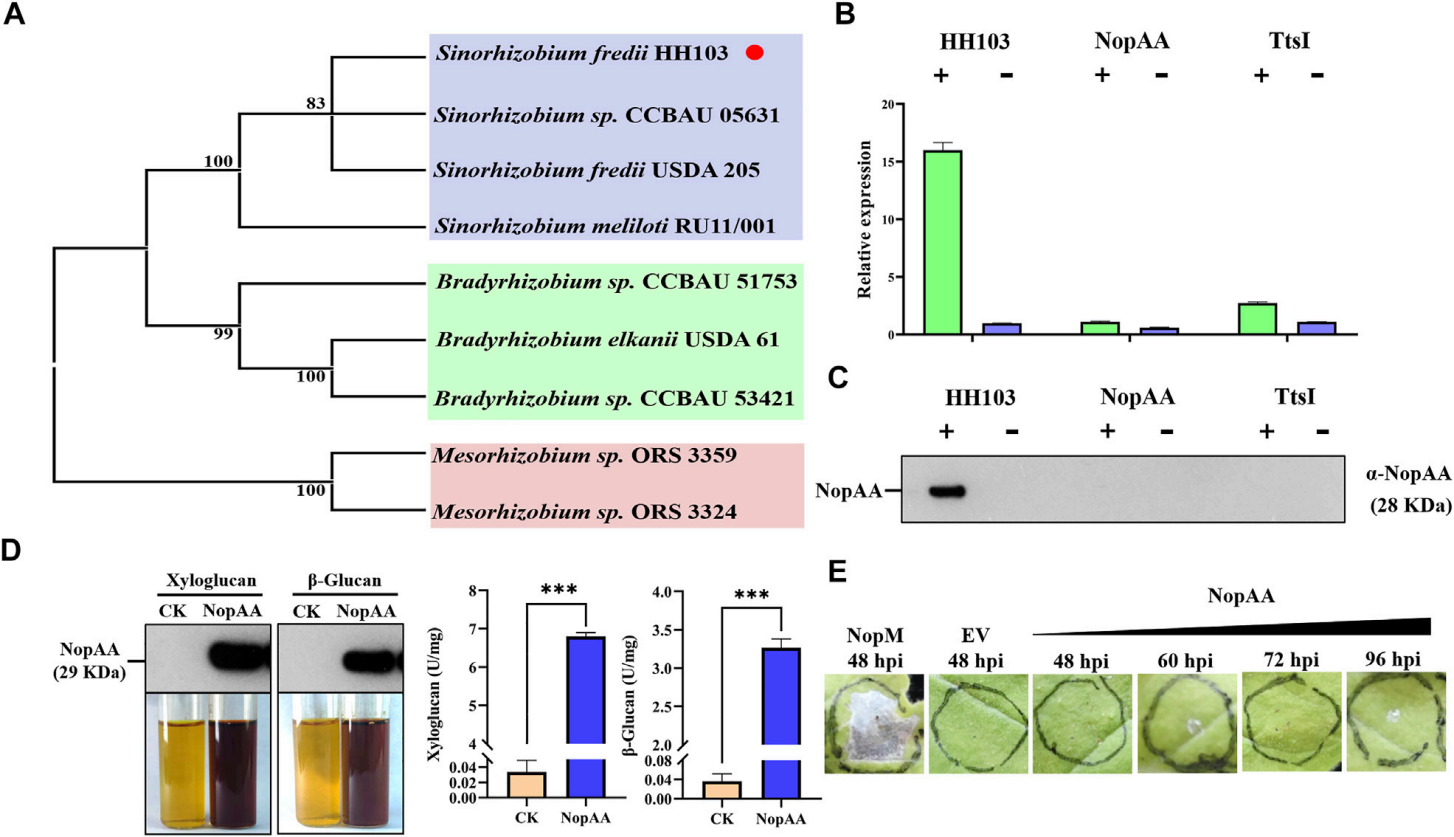
NopAA is a rhizobial type III effector with glycoside hydrolase activity. **(A)** Phylogenetic tree analysis of NopAA proteins from different rhizobia. **(B)** Relative expression of NopAA in rhizobia in the presence and absence of genistein. **(C)** Analysis of NopAA, a nodulation-promoting outer protein secreted by rhizobia, by immunoassay in the presence and absence of genistein. In **(B)** and **(C)**, "+" represents induction by the addition of genistein, and "−" represents the absence of genistein. **(D)** Analysis of glycoside hydrolase activity of NopAA. Pure xyloglucan and β-glucan were used as substrates for measuring enzyme activity, and NopAA protein in the reaction system was detected by immunoblotting. Data are presented as the average of three different biological replicates. **(E)** *N. benthamiana* leaves 48 h–96 h after inoculation with EH105 carrying *NopAA* and positive control *NopM*.

A phylogenetic analysis was performed to study the evolutionary relationships of NopAA in different rhizobial species. This showed that the NopAA tree was separated into three branches (blue, green, and pink regions in Figure 1A). The blue region contained only four *Sinorhizobium* species, the green region contained only three *Bradyrhizobium* species, and the pink region contained only two *Mesorhizobium* species (Figure 1A). The phylogenetic analysis showed that there is obvious diversity in NopAA among different rhizobial genera. Previous RNA-seq results showed that NopAA might be induced by genistein; thus, to further study whether NopAA was a type III effector, qRT-PCR and nodulation analyses were performed. The qRT-PCR results showed that genistein increased NopAA expression significantly in HH103 compared with rhizobia not treated with genistein but did not induce NopAA expression in the NopAA mutant or the TtsI mutant regardless of the presence or absence of genistein (Figure 1B). After extraction of the nodulation outer proteins, NopAA was detected in the supernatants after genistein treatment but not in the supernatants of the NopAA mutant or TtsI mutant outer proteins induced by genistein (Figure 1C). These results indicated that NopAA could be induced by genistein and TtsI and could be secreted into the extracellular milieu of the rhizobia.

### NopAA has Glycosyl Hydrolase Activity and Does not Induce Cell Death in Tobacco

To determine whether NopAA had glycosyl hydrolase activity, 6 × His-tagged NopAA was expressed and purified by Ni-affinity chromatography. The molecular weight of the His-NopAA fusion protein was ∼28 kDa (Figure 1D). Xyloglucan and β-glucan were used as substrates to analyze hydrolase activity, showing that NopAA hydrolyzed both xyloglucan and β-glucan directly to sugars (Figure 1D). The *Phytophthora sojae* apoplastic effector PsXEG1 and NopAA belong to the glycosyl hydrolase family, which is known to induce cell death in tobacco leaves. To study the effect of NopAA on tobacco leaves, we infiltrated NopAA into tobacco leaves using *Agrobacterium tumefaciens* EHA105. In contrast to PsXEG1, NopAA did not trigger cell death (Figure 1E). These results indicated that NopAA had glycosyl hydrolase activity and did not induce cell death in tobacco.

### NopAA Mutant Does not Promote Nodulation in Several Soybean Germplasms

In this study, we selected 13 soybean germplasms to study the effect of NopAA on nodulation, including five cultivated, six landrace soybean, and two wild soybean germplasms (Supplementary Figure S1). Nodulation tests showed that while the NopAA mutant significantly reduced the nodule numbers in 11 of the germplasms, the nodule numbers did not differ significantly in Dongnong594 (DN594) and Qingdou compared with the wild strain HH103. Inoculation with the NopAA mutant led to lower nodule dry weights in 12 of the 13 germplasms except for DN594, compared with the wild strain HH103. Nodulation tests of DN594 and Qingdou showed that the NopAA mutant did not influence nodule numbers, nor did it alter the change in the dry weight of DN594. These results indicated that NopAA did not negatively influence symbiosis in different types of soybean germplasms, and that differences in the genetic backgrounds of the germplasms might be the explanation for the variations in nodule traits associated with NopAA. This suggested that these genetic differences could be used to identify genes or loci associated with symbiosis.

### The Influence of NopAA on Dongnong594 and Charleston

**FIGURE 2:**
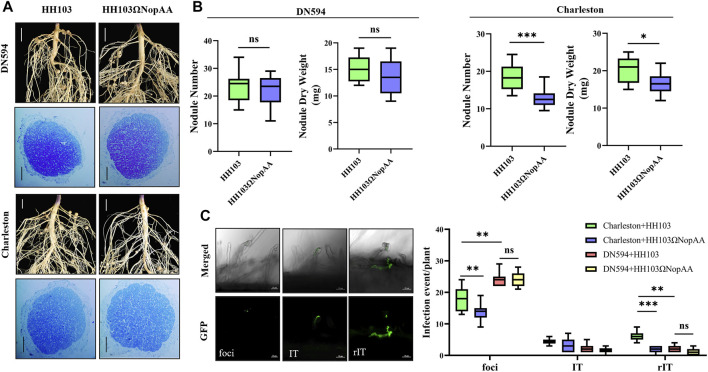
Differences in infection events between DN594 and Charleston after inoculation with HH103 or the NopAA mutant. **(A)** Phenotypes of DN594 and Charleston inoculated with HH103 and HH103ΩNopAA; root phenotype scale bars represent 0.5 cm; toluidine blue staining of nodules scale bars represent 50 μm. **(B)** Boxplots of NN and NDW. **(C)** Boxplots of the total number of infection events per plant of Charleston and DN594 (1 dpi). Data are presented as the average of three different biological replicates and ten root segments for each replicate, and all results were analyzed for significance using *t*-tests. Foci, infection foci; IT, infection thread in an epidermal cell; rIT, infection thread extending into a cortex cell. dpi, day post-inoculation.

Based on the results of the nodule tests on various soybean germplasms, DN594 and Charleston were selected for further investigation of the role of NopAA in symbiosis. Compared with the wild strain HH103, the NopAA mutants caused a significant reduction in both the nodule number (NN) and nodule dry weight (NDW) in Charleston but did not affect DN594 (Figures 2A,B). In the mature nodule, the infection zone and infection cells did not differ significantly between the two germplasms after inoculation with HH103, and inoculation with the NopAA mutant did not change this (Figure 2A). Because of the differences in the nodule traits of DN594 and Charleston, we further investigated the infection events after inoculation with GFP-labeled HH103 and the NopAA mutant. The results showed there were more infection events in curled root hairs in Charleston compared with DN594 3 days after inoculation with GFP-labeled HH103 compared with the wild strain, while the GFP -labeled NopAA mutant induced fewer infection events in Charleston but had no significant effects on DN594 (Figure 2C). This implied that the type III effector NopAA might influence nodulation through involvement in infection events and that the genetic differences between DN594 and Charleston might result in different symbiotic phenotypes, with some of the genetic differences being associated with NopAA.

### Quantitative Trait Locus for Nodule Number and Nodule Dry Weight Related to NopAA in Soybean Recombinant Inbred Line Populations

The soybean RILs were derived from the cross and continuous self-cross of DN594 and Charleston and were used to identify genes related to NopAA. Nodulation tests were performed on 150 RILs after inoculation with the wild strain and the NopAA mutant, with the results showing that the NopAA mutant could significantly reduce both the NN and NDW in whole RILs compared with HH103, further suggesting that NopAA plays a positive role in symbiosis. WinQTL Cartographer was used to identify the QTLs underlying nodule-related traits using a composite interval mapping method. Two main-effect QTLs underlying NDW and five QTLs underlying NN were identified with the background inoculated with *S. fredii* HH103 (Table. 1). Two QTLs, *QNDW3-1* and *QNDW7-1* were associated with NDW and explained 4.40% and 2.42% of the phenotypic variation, respectively. Five QTLs, *QNN9-1, QNN13-1, QNN13-2, QNN16-1,* and *QNN17-1.* were associated with NN, explaining 1.84%, 0.99%, 0.39%, 5.89%, and 6.13% of the phenotypic variation, respectively. A total of 33 candidate genes were found in these seven loci (Supplementary Table S3). In the background inoculated with the *S. fredii* NopAA mutant*,* two QTLs underlying NDW were located on chromosome 19, while only one QTL underlying NN was identified on chromosome 19 (Table 2). The QTLs *QNDW19-1* and *QNDW19-2* explained 0.15% and 2.59% of the phenotypic variation, respectively QTLs *QNDW19-2* and *QNN19-1* were in the same region, with very small R^2^ values. Twenty genes were identified in these loci (Supplementary Table S3). As no comparable region was identified in the backgrounds of *S. fredii* HH103 and the *S. fredii* NopAA mutant and as *QNDW19-2* and *QNN19-1* were in the same region, we considered that this QTL region might contain the key candidate genes for interaction with NopAA. Gene functional annotation showed that gene *Glyma.19G074000* encoded a nodulin protein, while *Glyma.19G073900* encoded a DNA lyase, and *Glyma.19G074200* was co-expressed with genes in the root-specific co-expression subnetwork. *Glyma.19G073800* and *Glyma.19G074100* had no specific functional annotations.

**TABLE 1:** Nodule traits in RILs inoculated with HH103 and HH103ΩNopAA.

Strain	RILs (*n* = 150)				Parents (average)	
	Trait	Average	Standard deviation	Coefficient of variation	Charleston	DN594
HH103	NN	11.4	5.6	48.95	21.0 ± 4.0	31.6 ± 5.3
	NDW	14.0	17.6	125.71	12.3 ± 2.8	19.5 ± 2.1
NopAA mutant	NN	8.1*	5.9	62.50	14.3 ± 4.2**	29.9 ± 6.2
	NDW	6.2*	3.9	185.85	5.5 ± 1.7**	18.8 ± 3.4

Note: NN, nodule number; NDW, nodule dry weight. * indicates significant differences with different inoculations, *p* ≤ 0.05, ** indicates *p* ≤ 0.01.

**TABLE 2:** Main QTLs identified in the RIL.

Strain	Trait	QTL	Chr.	Start position	End position	LOD	R^2^(%)	Putative causal genes or QTLs identified in previous study
HH103	NDW	*QNDW6-1*	6	13,842,716	14,216,591	3.0	4.40	Nodule size 1–3 Hwang et al. (2013)
		*QNDW7-1*	7	5, 985, 880	6, 014, 687	2.7	2.42	
	NN	*QNN9-1*	9	36, 047, 415	40, 663, 169	3.3	1.84	
		*QNN11-1*	11	30,891,632	33,719,971	2.8	0.99	Nodule weight per plant, dry 1–9 Nicol á s et al. (2006)
		*QNN13-1*	13	8, 930, 546	9, 544, 895	2.4	0.39	
		*QNN16-1*	16	8, 511, 616	9, 216, 892	3.7	5.89	
		*QNN17-1*	17	32, 105, 983	32, 555, 254	3.5	6.13	
NopAA mutant	NDW	*QNDW19-1*	19	19, 068, 552	19, 533, 854	3.3	0.15	
		*QNDW19-2*	19	25, 922, 562	27, 078, 098	3.9	2.59	
	NN	*QNN19-1*	19	25, 922, 286	27, 078, 098	3.6	4.19	

Note: Chr., chromosome; LOD, log-of-odds; QTL, quantitative trait loci; LOD score cutoff of major QTLs was determined by permutation tests (1,000 times; *p* < 0.05).

### qRT-PCR Verification of Candidate Genes

**FIGURE 3:**
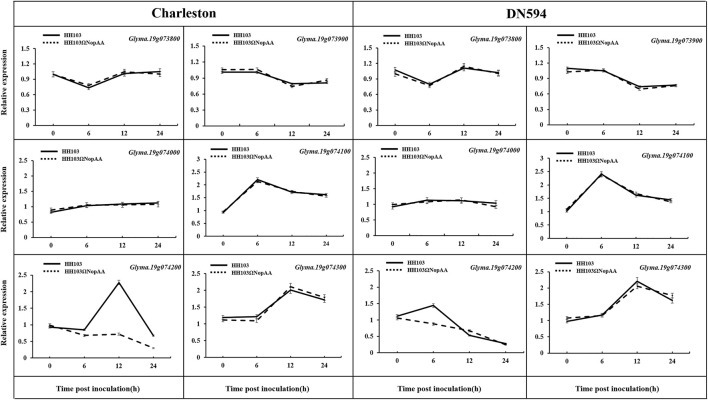
Analysis of the relative expression levels of candidate genes in the QTL interval by qRT-PCR. Relative expression of genes was calculated by the 2^−ΔΔCt^ method, and the relative expression levels of each gene were normalized using *GmUNK1* (*Glyma.12g020500*). Data are means ± SE of three replicates.

The candidate genes were verified by qRT-PCR to confirm their association with NopAA, using the roots of both DN594 and Charleston, as well as non-inoculated roots (Figure 3). Among these genes, *Glyma.19G074200* was confirmed to be upregulated with an almost two-fold change after inoculation with *S. fredii* HH103 but not with the *S. fredii* NopAA mutant 6 h after inoculation*.* This supported the association between *Glyma.19G074200* (named *GmARP*) and NopAA. The remaining genes were not significantly affected by the wild-type and mutant rhizobial inoculations.

### The Effects of *GmARP* Silencing and Overexpression on Nodulation

**FIGURE 4:**
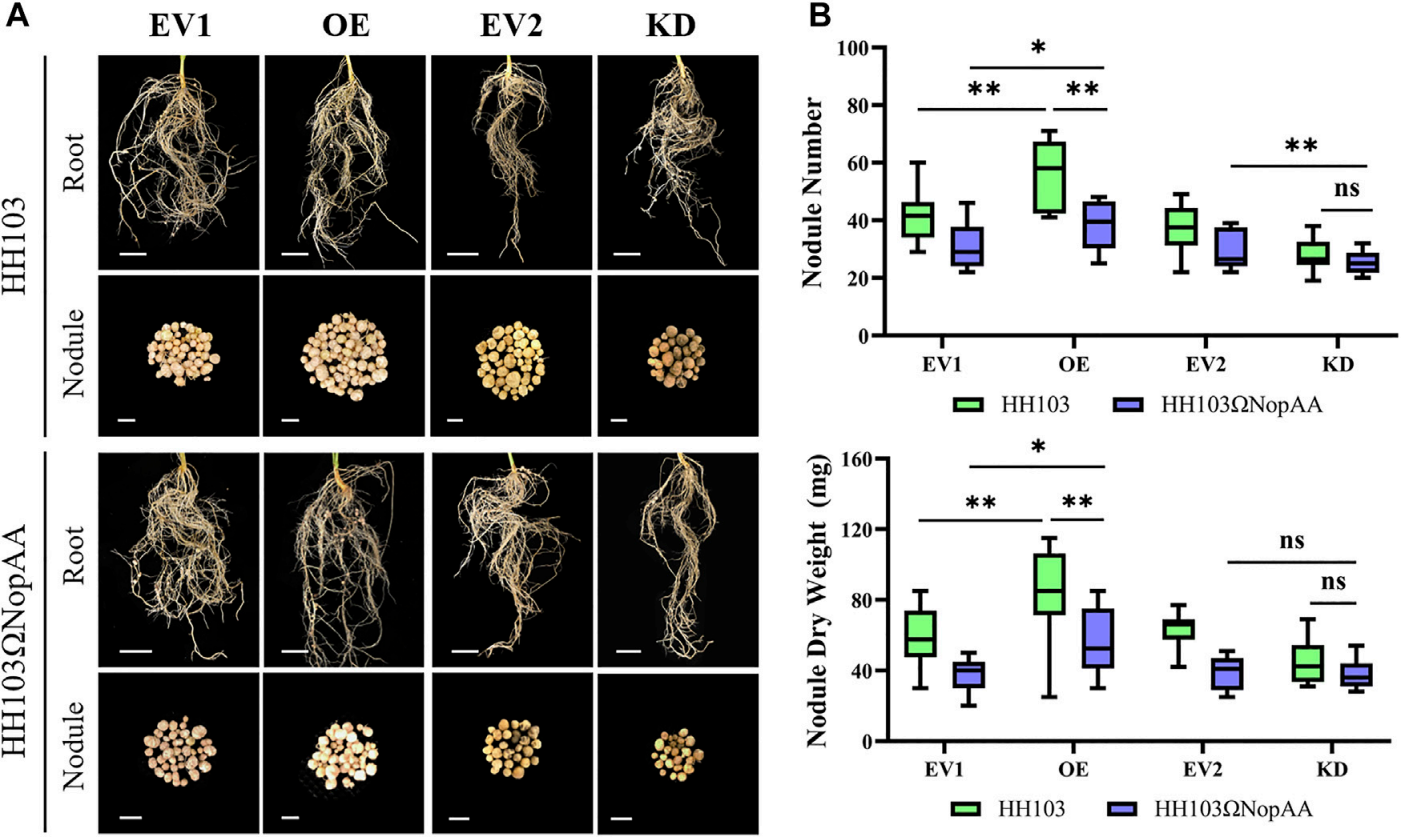
Nodule phenotypes of EV1, OE, and KD. **(A)** Nodular phenotypes of hairy roots transformed with EV1, OE, EV2, and KD after inoculation with HH103 and HH103ΩNopAA (28 dpi). EV1, Empty vector for gene overexpression; OE, Overexpression of *GmARP*; EV2, Empty vector for gene silencing; KD, Gene silencing of *GmARP* by RNAi. Root: scale bars represent 1 cm; nodule: scale bars represent 2 mm. **(B)** Boxplots of nodule phenotypes. Data are presented as the average of three different biological replicates and 15 plants for each replicate and significance was determined by *t*-tests.

To further investigate the role of *GmARP* in soybean nodulation, the *Agrobacterium rhizogenes* strain K599 carrying pB7GWIWG2(II)-*Dsred*-*GmARP* (for RNA interference), pSoy10-*GmARP-GFP* (for overexpression), and the corresponding empty vectors were used for the transformation of soybean transgenic hairy roots. Silencing and overexpression were confirmed by qRT-PCR after transformation (Supplementary Figure S2). Reduced NN and NDW values were seen after *GmARP* silencing compared with the controls inoculated with HH103 and the NopAA mutant, respectively (Figure 4A). In the silenced hairy roots, the NopAA mutant did not induce significantly different NN or NDW compared with HH103 (Figure 4B). In the overexpressed hairy roots, *GmARP* overexpression resulted in increased NN and NDW values compared with the controls inoculated with HH103 and the NopAA mutant, respectively (Figure 4B). We found that overexpression of *GmARP* led to elevated NN and NDW in comparison with the control hairy roots inoculated with the NopAA mutant. However, both the NN and NDW after inoculation with the NopAA mutant were still lower than HH103 in the overexpressed hairy roots, suggesting that overexpression could partially compensate for the loss of NopAA in HH103 during symbiotic formation. These results indicated that *GmARP* may be involved in signal transduction after recognition of NopAA by the soybean, leading to the regulation of nodulation.

### Haplotype Analysis Suggests That *GmARP* Regulates Nodule Number and Nodule Dry Weight in Soybean

**FIGURE 5:**
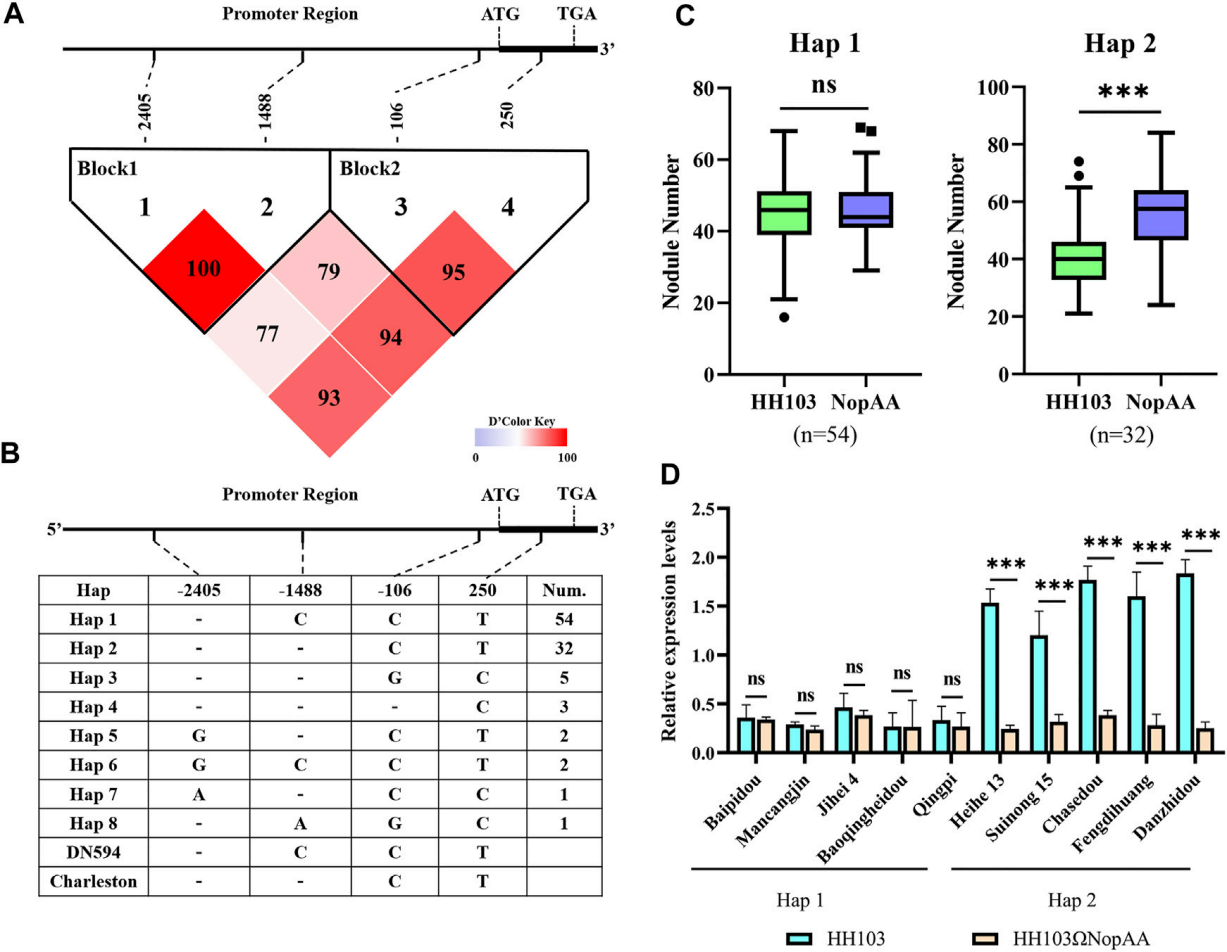
Haplotype analysis of *GmARP*. **(A**,**B)** Haplotype analysis of *GmARP* from 100 soybean resources. **(C)** NN of Hap1 and Hap2 inoculated with HH103 and HH103ΩNopAA, data are presented as the average of three different biological replicates and 15 plants for each replicate. **(D)** Gene expression of *GmARP* of Hap1 and Hap2 inoculated with HH103 and HH103ΩNopAA (12 hpi). Significance was measured by *t*-tests. NN, nodule number.

Based on the observed differences in the recognition of NopAA, haplotype (Hap) analysis of *GmARP* was performed in 100 soybean natural varieties using Dnasp5.0 software. Using the results of the resequencing of soybean natural varieties, eight Haps were identified. Two Haps containing more than 10 accessions were considered the dominant Haps, and only one SNP and one indel were observed in the promoter regions of Hap1 and Hap2 (Figures 5A,B). What is even more remarkable is that there was no difference in the NN and NDW values in the Hap1 accessions inoculated with HH103 compared with the NopAA mutant; however, in the Hap2 accessions, the NopAA mutant induced both lower NN and NDW than HH103 (Figure 5C). To identify whether the difference in the promoter region caused the expression difference between Hap1 and Hap2, qRT-PCR was used to analyze the expression of *GmARP* at 12 h post-inoculation with HH103 and the NopAA mutant in some varieties of Hap1 and Hap2. The expression patterns suggested that the relative expression level of *GmARP* did not differ between several Hap1 accessions (Heihe13 and Suinong15) regardless of the presence of NopAA in HH103 (Figure 5D). However, in the Hap2 accessions, HH103 induced significant expression of *GmARP* compared with the NopAA mutant. The haplotype analysis further confirmed that *GmARP* was associated with the type III effector NopAA to regulate nodulation in soybean.

## Discussion

In this study, we confirmed that NopAA was a type III effector and could hydrolyze xyloglucan and β-glucan directly into glucose. Nodulation tests indicated that NopAA did not have a negative effect on nodulation, and the soybean gene *GmARP* was found to be associated with NopAA in the soybean RIL population. There are few studies on NopAA function and the mechanisms involved, and few address the question of its role in symbiosis. Our study of NopAA provides an essential foundation for understanding the role of NopAA and its associated signaling pathways in symbiotic nitrogen fixation.

The type III effectors of rhizobia have obvious characteristics, that is, they are induced and regulated by flavonoids and TtsI, and the most important is that they can be secreted into the extracellular of rhizobia (Teulet et al., 2022). Similar to the reported type III effectors, such as NopD, NopL and NopM (Zhang et al., 2011b; Xin et al., 2012; Wang et al., 2020a), NopAA can also be induced by flavonoids and its expression can be regulated by TtsI, these confirmed NopAA is a type III effector. In the previous study, several type III effectors can act as enzymes and have biochemical activities (Teulet et al., 2022), and elucidating the biochemical functions of type III effectors, particularly those that are conserved among different species, has greatly enhanced our understanding of the mechanisms underlying bacterial pathogenesis (Chen et al., 2021; Zboralski et al., 2022). By analysis of NopAA amino acid sequence, we found that NopAA belonged to the Glycoside hydrolase 12 (GH12) family and had the potential to hydrolyze polysaccharides (Zhang et al., 2021b). Nodulation requires the assistance of cell-wall degrading enzymes, such as hemicellulases, pectinases, polygalacturonases, glucanases, cellulases, xyloglucanases, and pectinases, which allow root penetration by beneficial microorganisms, such as *Rhizobium, Frankia*, and the arbuscular mycorrhizal (Mateos et al., 1992; Zhang et al., 2021b; Tsyganova et al., 2021). GH12 is a cell wall-degrading enzyme that, along with the bacterial effector Cif and the *Phytophthora sojae* apoplastic effector PsXEG1, have been identified as playing pivotal roles in pathogen survival and systemic infection processes (Ma et al., 2015; Xia et al., 2020). Identifying the host protein interactors could elucidate the mechanisms underlying host-microbe interactions. NopAA was identified as a glycoside hydrolase belonging to the GH12 family. In *Phytophthora sojae*, PsXEG1 is also a glycoside hydrolase 12 family member with glycoside hydrolase activity and hydrolases the same substrates as NopAA. Because PsXEG1 is an effector of *P. sojae*, the replication of a *P. sojae* virus could be promoted using a PsXLP1 decoy that was similar to PsXEG1 but without its enzymatic activity. Additionally, PsXLP1 can protect PsXEG1 from GmGIP1 binding *in vitro* and *in planta* (Ma et al., 2015; Ma et al., 2017). We did not find any proteins similar to NopAA in the *S. fredii* HH103 genome, suggesting that HH103 does not infect soybeans using the same decoy pattern as *P. sojae* by NopAA. The investigation of the effect of NopAA on tobacco leaves did not show any evidence of cell death. Rhizobia may be involved in soybean immune signaling pathways during infection and the establishment of the symbiotic relationship. However, the establishment of an effective symbiosis does not require as strong or prolonged an immune response as occurs against pathogens. These results differ significantly from the actions of PsXEG1 in tobacco leaves. Thus, we propose that the role of NopAA may be different from that of PsXEG1. In this study, xyloglucan and β-glucan were found to be hydrolyzed to glucose; both xyloglucan and β-glucan are important components of cellulose and hemicellulose in plant cell walls, and their hydrolysis could promote the entry of rhizobia into host cells. These results are consistent with the observations of infection sites as fewer infection sites were observed with the NopAA mutant, indicating that NopAA could degrade the cell walls of soybean root hair cells, thus promoting infection and the formation of the infection thread.

A total of 10 QTLs were identified in this study, two of which were related to NDW, and five were related to NN with HH103, with two QTLs related to NDW and only one QTL related to NN seen with the NopAA mutant. No overlapping QTL loci were found when comparing inoculated HH103 and NopAA mutant, because NopAA mutation obviously changed the NN and NDW of DN594, Charleston and RIL populations, so no overlapping QTL could be identified, this result was similar to previous studies of other effectors, such NopL and NopT (Liu et al., 2021; Ni et al., 2022). Of the 10 identified QTLs, two were found to overlap with the reported symbiotic loci *Ns1-3* (Hwang et al., 2013) and *Nw1-9* (Nicol á s et al., 2006), respectively. These results indicated the accuracy of the QTL mapping and genetic analyses in the DN594 and Charleston RIL populations. The NopAA mutant was derived from the wild strain HH103 and was found to induce fewer NNs and lower NDW, as well as to generate fewer infection sites. Analysis of the expression patterns of several symbiotic marker genes showed that the NopAA mutant could also change the expression pattern of genes, such as PR1. It is possible that the interaction network might change without the involvement of NopAA. These results suggested that the soybean response to the NopAA mutant differed significantly from its response to the wild strain, leading to the observed QTL differences between HH103 and the NopAA mutant.

In the overlapped QTLs, *Glyma.19G074200* was found to be associated with NopAA using gene annotation and qRT-PCR. *Glyma.19G074200* is a root-specific co-expression gene belonging to the uncharacterized protein At3g27210 protein family. In *Arabidopsis*, the At3g27210 protein interacts with AtRKL1 (Tarutani et al., 2004) and participates in the response to pathogen infection. Soybean RNA-seq results confirmed that *Glyma.19G074200* was involved in symbiosis during *Bradyrhizobium japonicum* infection, although the function of the gene was not determined (Libault et al., 2010). In soybean, the function of this gene has not been studied, in this study, overexpression of *GmARP* promoted nodule formation after inoculation with either the wild strain or the NopAA mutant but, after *GmARP* silencing, no significant differences in either NN or NDW between plants inoculated with HH103 or the NopAA mutant were observed. These results suggest that *GmARP* is a positive regulator of nodule formation and that it mediates NopAA signaling in plants, the mechanism of the synergistic regulation of *GmARP* and NopAA in symbiosis remains to be further studied. We demonstrated that NopAA had glycosyl hydrolase activity and may promote rhizobia infection by hydrolyzing the host cell wall, and that host recognition of NopAA activated the expression of symbiotic genes. When HH103 was used for infection, NopAA induced *GmARP* expression to complete the establishment of symbiosis. We have two hypotheses concerning the mechanism of this activated expression: the first is that NopAA induces the expression of *GmARP* by activating its downstream signals while the second is that the products resulting from NopAA hydrolysis of cell wall polysaccharides induce signaling changes in the host cells, which further activate the expression of *GmARP*. However, these hypotheses require further experimental verification.

## Conclusion

We demonstrated that NopAA is a type III effector with glycosyl hydrolase activity. Investigation of the effects of the NopAA mutant on nodulation showed that the mutant did not promote nodulation. Using QTL mapping and analysis of soybean genes associated with NopAA, we found that the soybean protein GmARP positively regulated nodulation through its association with NopAA. This study provides an essential reference for analyzing the function of NopAA and provides support for efficient utilization of symbiotic nitrogen fixation in soybean agricultural production.

## Data Availability

The original contributions presented in the study are included in the article/Supplementary Material, further inquiries can be directed to the corresponding authors.

## Supplementary Material

Supplementary Figure S1 Identification of nodulation ability of soybean germplasm resources. **(A)** Boxplot of NNs of soybean varieties inoculated with HH103 or HH103ΩNopAA (28 dpi). **(B)** Boxplot of NDWs of soybean varieties inoculated with HH103 or HH103ΩNopAA (28 dpi). Data are presented as the average of three different biological replicates and 15 plants for each replicate, results were analyzed for significance using the *t*-test. NDW, nodule dry weight; NN, nodule number.

Supplementary Figure S2 Relative expression levels of *GmARP* in transformed hairy roots. Data are means  ±  SE.
