# Supplementary figures and images for: GmARP is Related to the Type III Effector NopAA to Promote Nodulation in Soybean (Glycine max)
Source: Front Genet. 2022 May 27;13:889795. doi: 10.3389/fgene.2022.889795 (PMC9184740; doi:10.3389/fgene.2022.889795)

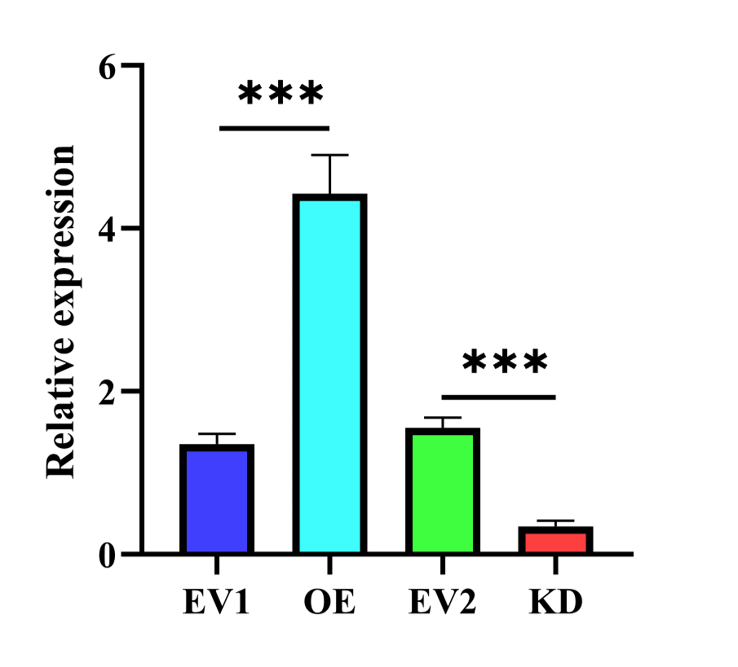

Supplement: Supplementary file 1 [file Image2.TIF]

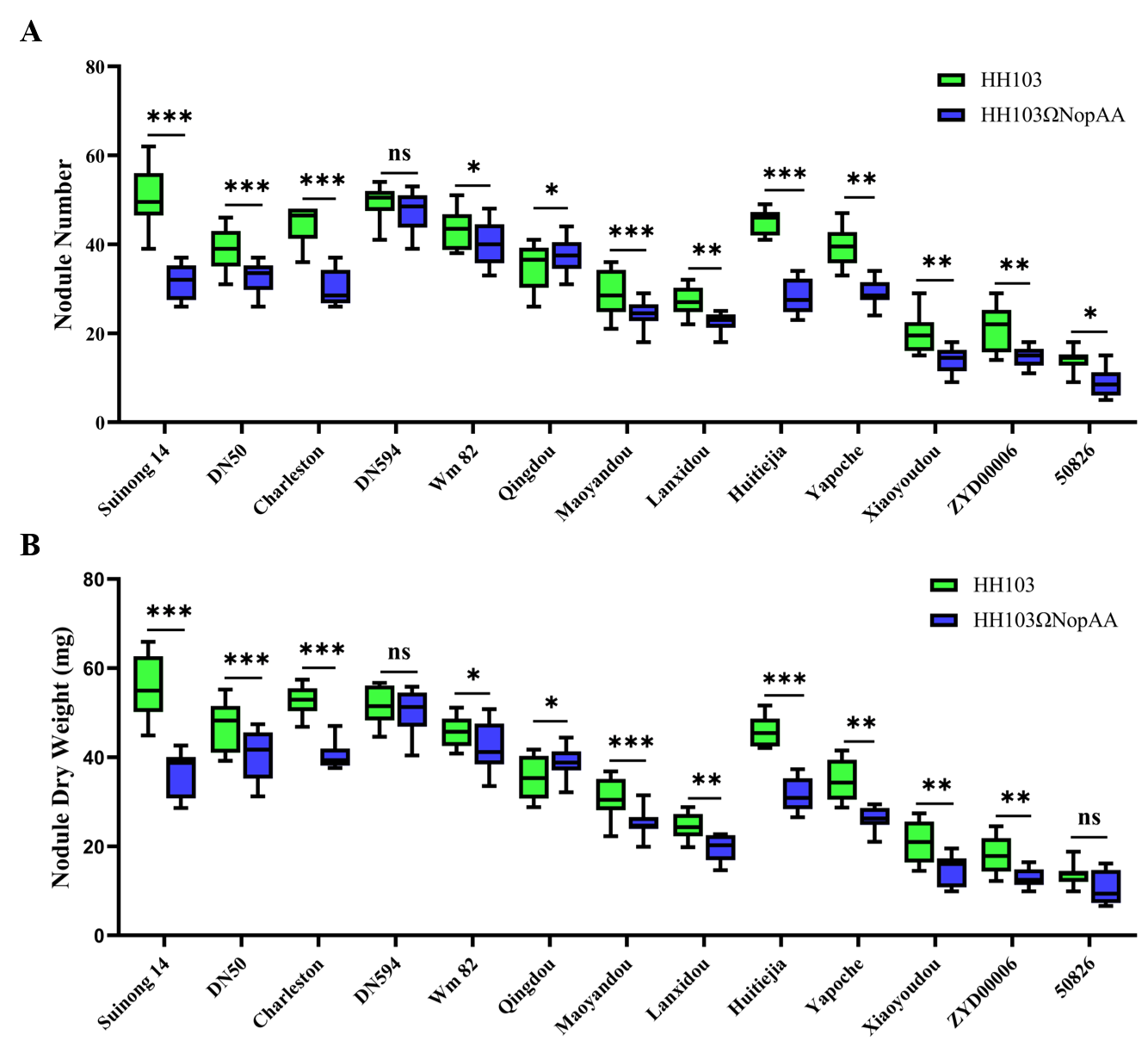

Supplement: Supplementary file 2 [file Image1.TIF]
