# Supplementary material for: GmARP is Related to the Type III Effector NopAA to Promote Nodulation in Soybean (Glycine max)
Source: Front Genet. 2022 May 27;13:889795. doi: 10.3389/fgene.2022.889795 (PMC9184740; doi:10.3389/fgene.2022.889795)
Supplement: Supplementary file 3 [file DataSheet1.docx]

**Table S1.** Information of Primers Used

| **No.** | **Name of primer** | **Primer 5’-3’** | **Annotation and Functions** |
| --- | --- | --- | --- |
| 1 | pET28b-NopAA-F | CCGgaattcAATGGATTCCAATCGGATAAA | Expression of NopAA, in *E.coli* BL21 for purification |
| 2 | pET28b-NopAA-R | ACGCgtcgacCTTCGAGGTCACAGACCAGT | Expression of NopAA, in *E.coli* BL21 for purification |
| 3 | pGWC-NopAA-F- | ATGGATTCCAATCGGATAAAC | Clone *NopAA* of *Sinorhizobium fredii* HH103 |
| 4 | pGWC-NopAA-R- | TCACTTCGAGGTCACAGACCAG | Clone *NopAA* of *Sinorhizobium fredii* HH103 |
| 5 | pGWC-NopAA1.4-F- | TGAAGAACGATTGGTGAACG | Clone the fragment containing *NopAA* and putative promoter intio pGWC |
| 6 | pGWC-NopAA1.4-R- | GTCGCAACAGGGTTAGCCAG | Clone the fragment containing *NopAA* and putative promoter intio pGWC |
| 7 | pGWC-NopAA1.4-mutant-F- | TATGGATTCCAATCGactagtCGGTGGCGCCGCGAA | Site-directed mutagenesis, mutated *NopAA*1.4 had a *Spe1* restriction site |
| 8 | pGWC-NopAA1.4-mutant-R- | TTCGCGGCGCCACCGactagtCGATTGGAATCCATA | Site-directed mutagenesis, |
| 9 | Kan-*Spe1*-F | actagtAGTAAACTGGATGGCTTTCTTG | Clone Kanamycin fragment into pGWC-NopAA1.4 |
| 10 | Kan-*Spe1*-R | actagtCTTCAGCATCTTTTACTTTCAC | Clone Kanamycin fragment into pGWC-NopAA1.4 |
| 11 | pJQ200SK-NopAAΩ-F | GCtctagaTGAAGAACGATTGGTGAACG | Construction of Suicide vector pJQ200SK-NopAAΩ to generate HH103Ω*NopAA* |
| 12 | pJQ200SK-NopAAΩ -R | ACGCgtcgacGTCGCAACAGGGTTAGCCAG | Construction of Suicide vector pJQ200SK-NopAAΩ to generate HH103Ω*NopAA* |
| 13 | qRT- 19G072600-F | ATGCAAACCCCTGAAAGT | qRT-PCR for validation of Gm19g072600 gene transcription level in Charleston and DN594 |
| 14 | qRT- 19G072600-R | GAGATTTTGT TTGCTCCGC | qRT-PCR for validation of Gm19g072600 gene transcription level in Charleston and DN594 |
| 15 | qRT- 19G072700-F | ATGAAGCTTTCAGATGATG | qRT-PCR for validation of Gm19g072700 gene transcription level in Charleston and DN594 |
| 16 | qRT- 19G072700-R | ACAGTTTCTCGTAAAGCAG | qRT-PCR for validation of Gm19g072700 gene transcription level in Charleston and DN594 |
| 17 | qRT- 19G072800-F | TTGCAACCAATGGAGAGATTTG | qRT-PCR for validation of Gm19g072800 gene transcription level in Charleston and DN594 |
| 18 | qRT- 19G072800-R | GCTCCAGTTTTGAATTTGGACT | qRT-PCR for validation of Gm19g072800 gene transcription level in Charleston and DN594 |
| 19 | qRT- 19G072900-F | GAAAGCTGTGTTCTTCGGATAC | qRT-PCR for validation of Gm19g072900 gene transcription level in Charleston and DN594 |
| 20 | qRT- 19G072900-R | GCCCAAGTTTAGACTCATTTGG | qRT-PCR for validation of Gm19g072900 gene transcription level in Charleston and DN594 |
| 21 | qRT- 19G073000-F | CAATGAAATGGCTTCTCGTCAT | qRT-PCR for validation of Gm19g073000 gene transcription level in Charleston and DN594 |
| 22 | qRT- 19G073000-R | CTCTCCCTTTTGCAAAGCTTAG | qRT-PCR for validation of Gm19g073000 gene transcription level in Charleston and DN594 |
| 23 | qRT- 19G073100-F | ACAAAAATACCCTATGGCTCCA | qRT-PCR for validation of Gm19g073100 gene transcription level in Charleston and DN594 |
| 24 | qRT- 19G073100-R | GCCATAGATTTGGGTTCGTAAC | qRT-PCR for validation of Gm19g073100 gene transcription level in Charleston and DN594 |
| 25 | qRT- 19G073200-F | ATGAACCCAAGACAACTCCTCC | qRT-PCR for validation of Gm19g073200 gene transcription level in Charleston and DN594 |
| 26 | qRT- 19G073200-R | GGGGTGTACATGAGAGTCAG | qRT-PCR for validation of Gm19g073200 gene transcription level in Charleston and DN594 |
| 27 | qRT- 19G073300-F | ATGGTGTTAGAAATGGTGG | qRT-PCR for validation of Gm19g073300 gene transcription level in Charleston and DN594 |
| 28 | qRT- 19G073300-R | TATGGTTTCAACATTCCCT | qRT-PCR for validation of Gm19g073300 gene transcription level in Charleston and DN594 |
| 29 | qRT- 19G073400-F | CCGAACCTCACTCTTTCAGATA | qRT-PCR for validation of Gm19g073400 gene transcription level in Charleston and DN594 |
| 30 | qRT- 19G073400-R | CAGAACACCCAATTATGTGCTC | qRT-PCR for validation of Gm19g073400 gene transcription level in Charleston and DN594 |
| 31 | qRT- 19G073500-F | GTGTGAAATTGTGCTAACCCTT | qRT-PCR for validation of Gm19g073500 gene transcription level in Charleston and DN594 |
| 32 | qRT- 19G073500-R | TAGGCACCAAAGACAAAAGTTG | qRT-PCR for validation of Gm19g073500 gene transcription level in Charleston and DN594 |
| 33 | qRT- 19G073600-F | ACAATAGTGGCATTACTTCCGA | qRT-PCR for validation of Gm19g073600 gene transcription level in Charleston and DN594 |
| 34 | qRT- 19G073600-R | ATAGTTGTGAGTTCGTTGCAAC | qRT-PCR for validation of Gm19g073600 gene transcription level in Charleston and DN594 |
| 35 | qRT- 19G073700-F | AGGGCGAGGTTTAACTATTGAA | qRT-PCR for validation of Gm19g073700 gene transcription level in Charleston and DN594 |
| 36 | qRT- 19G073700-R | CATCTTTGTCGCTCATTGTTGT | qRT-PCR for validation of Gm19g073700 gene transcription level in Charleston and DN594 |
| 37 | qRT-19g073800-F | CTTCTCCGTGGTCAGATTTG | qRT-PCR for validation of Gm19g073800 gene transcription level in Charleston and DN594 |
| 38 | qRT-19g073800-R | CATCTGAGAGACTGTCAACAAGC | qRT-PCR for validation of Gm19g073800 gene transcription level in Charleston and DN594 |
| 39 | qRT-19g073900-F | GTGAAGTGTGGCTATCTAAAAAA | qRT-PCR for validation of Gm19g073900 gene transcription level in Charleston and DN594 |
| 40 | qRT-19g073900-R | ATCATTTTCTCCCCGTATTCTTT | qRT-PCR for validation of Gm19g073900 gene transcription level in Charleston and DN594 |
| 41 | qRT-19g074000-F | ATCTTGTGCATAAAGGCAAATA | qRT-PCR for validation of Gm19g074000 gene transcription level in Charleston and DN594 |
| 42 | qRT-19g074000-R | TAAAAAAAAGAAGTAAAGCATGTC | qRT-PCR for validation of Gm19g074000 gene transcription level in Charleston and DN594 |
| 43 | qRT-19g074100-F | GTTGTGCCAGAGGTGTGAGA | qRT-PCR for validation of Gm19g074100 gene transcription level in Charleston and DN594 |
| 44 | qRT-19g074100-R | TGGTTTCTGAGTAGGTCTTGGT | qRT-PCR for validation of Gm19g074100 gene transcription level in Charleston and DN594 |
| 45 | qRT-19g074200-F | TATCCAAGAAGGTAAAGTAGTCG | qRT-PCR for validation of Gm19g074200 gene transcription level in Charleston and DN594 |
| 46 | qRT-19g074200-R | ATGAAGCAGTTATCCTAGATGTT | qRT-PCR for validation of Gm19g074200 gene transcription level in Charleston and DN594 |
| 47 | qRT-05g247300-F | TTGTCAGAGTCGGAGCCCATA | qRT-PCR for validation of Gm05g247300 gene transcription level in Charleston and DN594 |
| 48 | qRT-05g247300-R | GGAAGAAAAAGGGGGGAGATG | qRT-PCR for validation of Gm05g247300 gene transcription level in Charleston and DN594 |
| 49 | qRT-08g055700-F | CTAAATCCTGTGCGTAAGTTG | qRT-PCR for validation of Gm08g055700 gene transcription level in Charleston and DN594 |
| 50 | qRT-08g055700-R | GTATTGATTGGGAAGGAAAAG | qRT-PCR for validation of Gm08g055700 gene transcription level in Charleston and DN594 |
| 51 | qRT-UNK1-F | TGGTGCTG CCGCTATTTACTG | Reference gene for qRT-PCR |
| 52 | qRT-UNK1-R | GGTGGAAGGAACTGCTAACAAT | Reference gene for qRT-PCR |
| 53 | Fu28-ANP-F | ATGGATTCCAATCGGATAAAC | Clone *GmANP* fragment into Fu28 |
| 54 | Fu28-ANP-R | GGTACCCTTCGAGGTCACAGACCAG | Clone *GmANP* fragment into Fu28 |
| 55 | Fu28-NopAA-F | ATGGATTCCAATCGGATAAA | Clone *NopAA* fragment into Fu28 |
| 56 | Fu28- NopAA-R | GAATTC CTTCGAGGTCACAGACCAGT | Clone *NopAA* fragment into Fu28 |
| 57 | RNAi-ANP-F | GACCACCTTCACTGACCATG | Clone *GmANP-RNAi* fragment into pGWC for gene silencing |
| 58 | RNAi-ANP-R | TTTAGGCATAATAACATCTT | Clone *GmANP-RNAi* fragment into pGWC for gene silencing |
| 59 | qRT-NopAA-F | ATGGATTCCAATCGGATAAA | qRT-PCR for validation of NopAA gene transcription level |
| 60 | qRT-NopAA-R | gcgtgcgaactccaaatcgg | qRT-PCR for validation of NopAA gene transcription level |

**Table S2.** Information of Strains and Vectors

| **Strain** | **Relevant characteristics** | **Reference** |
| --- | --- | --- |
| ***Escherichia coli*** |  |  |
| DH5α | supE44 lacY169 (80lacZM15) hsdR17 recA1 endA1 gyrA96 thi-1 relA1 | Mason *et al*. 1989 |
| BL21(DE3) | F- ompT hsdSB (rB- mB-) gal dcm (DE3) | Studier *et al.* 1990) |
| ***Rhizobium* strains** |  |  |
| HH103 | Broad host range bacterium isolated from nodules of Glycine max , Rif^r^ | Dowdle *et al.* 1985 |
| HH103ΩNopAA | HH103 insertion mutated containing an Kanamycin resistance gene insertion at position downstream 17bp of start codon of *NopAA* nucleotide sequence, Rifr, Kan^r^ | This work |
| **Plasmids** |  |  |
| pGWC | Entry clone vector,Cm^r^ | Chen *et al*. 2006) |
| pET28b- NopAA | A 807-bp fragement of the *NopAA* coding region cloned into pET28b with primer1 and primer2 (Kan^r^) | This work |
| pJQ200SK | Suicide vector used for directed mutagenesis (Gm^r^) | Quandt and Hynes 1993 |
| pJQ200SK-NopAAΩ | A 2.4kb Xba1-Sal1 fragment containing *NopAA* with a Kanamycin resistance gene inserted into downstream 17bp of start codon of *NopAA* the Xba1-SalI site of pJQ200SK (Gm^r^) | This work |
| pRK2013 | Tra+ helper plasmid for mobilisation (Kan^r^) | Figurski and Helinski, 1979 |

Note: Rifampicin(Rif^r^ ), Kanamycin (Kan^r^), Chloramphenicol(Cm^r^), Gentamicin(Gm^r^)

**Table S3 The candidate genes in the located QTLs of wild type HH103 background**

| **Strain** | **Trait** | **List** | **Gene ID** | **Position** | **Domian/Function** |
| --- | --- | --- | --- | --- | --- |
| HH103 | NN | 1 | Glyma.11G227000 | 32195122..32196862 | Heat shock protein 90.1 |
|  |  | 2 | Glyma.11G220700 | 31603516..31604786 | Pathogenesis-related thaumatin superfamily protein |
|  |  | 3 | Glyma.11G222600 | 31769174..31771265 | Protein phosphatase 2CA |
|  |  | 4 | Glyma.11G227800 | 32282511..32283549 | WUSCHEL related homeobox 5 |
|  |  | 5 | Glyma.11G240900 | 33511429..33519903 | Receptor protein-tyrosine kinase |
|  |  | 6 | Glyma.13G029800 | 9110787..9114298 | Viral movement protein (MP) |
|  |  | 7 | Glyma.13G036000 | 11206450..11214565 | PTHR13856:SF95 - F24J8.3 protein |
|  |  | 8 | Glyma.13G036100 | 11279309..11284080 | Coexpressed with genes in leaf specific coexpression subnetwork |
|  |  | 9 | Glyma.16G082200 | 9017557..9025383 | PTHR10366:SF384 - NAD(P)-binding rossmann-fold superfamily Y protein |
|  |  | 10 | Glyma.16G082100 | 8889367..8889528 | Perakine reductase |
|  |  | 11 | Glyma.17G202700 | 32339585..32345876 | PTHR21319:SF15 - CHY AND CTCHY AND RING-TYPE ZINC FINGER PROTEIN |
|  |  | 12 | Glyma.17G202600 | 32331705..32334527 | PF04570 - zinc-finger of the FCS-type, C2-C2 (zf-FLZ) |
|  |  | 13 | Glyma.17G202800 | 32353449..32354610 | PTHR12228 - transcription initiation factor tfiid 55 KD subunit-related |
|  | NDW | 1 | Glyma.06G166800 | 13904476..13906745 | SWEET10 |
|  |  | 2 | Glyma.06G168400 | 14076804..14078036 | WRKY DNA-binding protein 75 |
|  |  | 3 | Glyma.06G169600 | 14167537..14172119 | Nuclear factor Y, subunit C1 |
|  |  | 4 | Glyma.06G169900 | 14190161..14191076 | Protein of unknown function (DUF579) |
|  |  | 5 | Glyma.07G066400 | 5985880..5986044 | PTHR12922 - ubiquinone biosynthesis protein |
|  |  | 6 | Glyma.07G066500 | 5987261..5990684 | PTHR31509:SF3 - bypass1-related protein-related |
|  |  | 7 | Glyma.07G066600 | 6000626..6003834 | PTHR27001:SF156 - protein kinase family protein |
|  |  | 8 | Glyma.07G066700 | 6012177..6014687 | Ubiquinone biosynthesis protein COQ4 |
| NopAAmutant | NN | 1 | Glyma.19G072600 | 25619825..25620195 | Unknown |
|  |  | 2 | Glyma.19G072700 | 25660955..25663777 | Cell division control protein 48 homolog a-related |
|  |  | 3 | Glyma.19G072800 | 25957072..25962027 | Ubiquitin-conjugating enzyme E2 S (UBE2S, E2EPF) |
|  |  | 4 | Glyma.19G072900 | 26101897..26103958 | Auxin efflux carrier family protein |
|  |  | 5 | Glyma.19G073000 | 26107043..26108643 | 60S ribosomal protein l18a // subfamily not named |
|  |  | 6 | Glyma.19G073100 | 26191061..26192340 | Oxidoreductase, 2og-fe ii oxygenase family protein // subfamily not named |
|  |  | 7 | Glyma.19G073200 | 26248452..26264052 | Wall-associated receptor kinase C-terminal (WAK_assoc) |
|  |  | 8 | Glyma.19G073300 | 26265382..26265708 | Glycerophosphoryl diester phosphodiesterase |
|  |  | 9 | Glyma.19G073400 | 26265784..26267956 | Wall-associated receptor kinase C-terminal (WAK_assoc) |
|  |  | 10 | Glyma.19G073500 | 26302984..26303190 | Unknown |
|  |  | 11 | Glyma.19G073600 | 26559317..26565444 | Nicotinamide adenine dinucleotide transporter 2, mitochondrial |
|  |  | 12 | Glyma.19G073700 | 26623876..26627989 | Myb/SANT-like DNA-binding domain (Myb_DNA-bind_3) |
|  |  | 13 | Glyma.19G073800 | 26814891..26815356 | Unknown |
|  |  | 14 | Glyma.19G073900 | 26847191..26847541 | DNA-(apurinic or apyrimidinic site) lyase / Phage-T4 UV endonuclease |
|  |  | 15 | Glyma.19G074000 | 26879967..26881966 | Nodulin-26B precursor |
|  |  | 16 | Glyma.19G074100 | 27004892..27005056 | Unknown |
|  |  | 17 | Glyma.19G074200 | 27018815..27022399 | Coexpressed with genes in roots specific coexpression subnetwork |
|  |  | 18 | Glyma.19G074300 | 27033857..27036326 | Unknown |
|  |  | 19 | Glyma.19G074400 | 27052958..27054492 | Unknown |
|  |  | 20 | Glyma.19G074500 | 27068157..27069844 | Surfeit locus protein 6 |
|  | NDW | 1 | Glyma.19G067400 | 19102309..19110451 | ADP-ribosylation factor gtpase-activating protein agd14-related |
|  |  | 2 | Glyma.19G067500 | 19342099..19345266 | Protein phytochrome kinase substrate 4 |

**Table S4 The haplotypes of 100 soybean natural varieties**

| No. | Name | Type | Haplotype |
| --- | --- | --- | --- |
| 1 | Baipidou | Landrace | Hap1 |
| 2 | Mancangjin | Landrace | Hap1 |
| 3 | Jihei 4 | Improved cultivar | Hap1 |
| 4 | Baoqingheidou | Landrace | Hap1 |
| 5 | Dongnong44 | Improved cultivar | Hap1 |
| 6 | Heihe 35 | Improved cultivar | Hap1 |
| 7 | Heihe 29 | Improved cultivar | Hap1 |
| 8 | Heihe 27 | Improved cultivar | Hap1 |
| 9 | Beifeng 11 | Improved cultivar | Hap1 |
| 10 | Heihe 11 | Improved cultivar | Hap1 |
| 11 | Hefeng 30 | Improved cultivar | Hap1 |
| 12 | Jiyu 67 | Improved cultivar | Hap1 |
| 13 | Hefeng 24 | Improved cultivar | Hap1 |
| 14 | Suinong 1 | Improved cultivar | Hap1 |
| 15 | Helongyoutai | Landrace | Hap1 |
| 16 | Kebei 1 | Improved cultivar | Hap1 |
| 17 | Fangzhengmoshidou | Landrace | Hap1 |
| 18 | Mengdou 12 | Improved cultivar | Hap1 |
| 19 | Hefeng 25 | Improved cultivar | Hap1 |
| 20 | Suinong 14 | Improved cultivar | Hap1 |
| 21 | Longquantaidou | Landrace | Hap1 |
| 22 | Liushitianhuancang | Landrace | Hap1 |
| 23 | Chasedou | Landrace | Hap1 |
| 24 | Jiunong 21 | Improved cultivar | Hap1 |
| 25 | Tiejiasilihuang | Landrace | Hap1 |
| 26 | Shidadou 1 | Landrace | Hap1 |
| 27 | Changjihuangdou | Landrace | Hap1 |
| 28 | Xindadou 1 | Landrace | Hap1 |
| 29 | Jinzhou 4-1 | Landrace | Hap1 |
| 30 | Dongnong 47 | Improved cultivar | Hap1 |
| 31 | Jiyu 71 | Improved cultivar | Hap1 |
| 32 | Tiefeng 8 | Improved cultivar | Hap1 |
| 33 | Dongda 2 | Improved cultivar | Hap1 |
| 34 | Huangqi | Landrace | Hap1 |
| 35 | Tianedan | Landrace | Hap1 |
| 36 | Tie 5621 | Landrace | Hap1 |
| 37 | Nonganpingding 4 | Landrace | Hap1 |
| 38 | Fusonghuangdadou | Landrace | Hap1 |
| 39 | Qunxuan 1 | Landrace | Hap1 |
| 40 | Shuangliaochadou | Landrace | Hap1 |
| 41 | Shulanshuilizhan | Landrace | Hap1 |
| 42 | Jiaohexiaolihuang | Landrace | Hap1 |
| 43 | Tonghuapingdinghuang | Landrace | Hap1 |
| 44 | Jiutaibaihuadou | Landrace | Hap1 |
| 45 | Baoqingheidou | Landrace | Hap1 |
| 46 | Neianxibeiwa | Landrace | Hap1 |
| 47 | Bolidalihuang | Landrace | Hap1 |
| 48 | Bailixiaoqihuang | Landrace | Hap1 |
| 49 | Qinganxiaojinhuang | Landrace | Hap1 |
| 50 | Xiaobaiqi | Landrace | Hap1 |
| 51 | Qinggangdali | Landrace | Hap1 |
| 52 | Liuyedou | Landrace | Hap1 |
| 53 | Jiajiedadou | Landrace | Hap1 |
| 54 | Xiaohuiqi | Landrace | Hap1 |
| 55 | Heihe 13 | Improved cultivar | Hap2 |
| 56 | Suinong 15 | Improved cultivar | Hap2 |
| 57 | Heihe 4 | Improved cultivar | Hap2 |
| 58 | Heinong 44 | Improved cultivar | Hap2 |
| 59 | Yantaohuangdou | Landrace | Hap2 |
| 60 | He Feng 51 | Improved cultivar | Hap2 |
| 61 | Qinanheidou | Landrace | Hap2 |
| 62 | Jilin 20 | Improved cultivar | Hap2 |
| 63 | Gongye 5056 | Landrace | Hap2 |
| 64 | Gongye 03-7239 | Landrace | Hap2 |
| 65 | Huangbaozhu | Landrace | Hap2 |
| 66 | Jiyu 66 | Improved cultivar | Hap2 |
| 67 | Nenfeng 11 | Improved cultivar | Hap2 |
| 68 | Jilin 30 | Improved cultivar | Hap2 |
| 69 | Baichengmoshidou | Landrace | Hap2 |
| 70 | Heimoshidou | Landrace | Hap2 |
| 71 | Niumaohuang | Landrace | Hap2 |
| 72 | Panshidou | Landrace | Hap2 |
| 73 | Tailaisilihuang | Landrace | Hap2 |
| 74 | Qianguosihualanqi | Landrace | Hap2 |
| 75 | Shuangyanhanhuangdou | Landrace | Hap2 |
| 76 | Zhaozhouxiaolihuang | Landrace | Hap2 |
| 77 | Yonglihuang | Landrace | Hap2 |
| 78 | Wuchangdou | Landrace | Hap2 |
| 79 | Qishibaimei | Landrace | Hap2 |
| 80 | Huangzhongge | Landrace | Hap2 |
| 81 | Silihuang | Landrace | Hap2 |
| 82 | Jiutaizhuyandou | Landrace | Hap2 |
| 83 | Bolibaihuacuo | Landrace | Hap2 |
| 84 | Ji 101 | Improved cultivar | Hap2 |
| 85 | Jifeng 2 | Improved cultivar | Hap2 |
| 86 | Jiyu 92 | Improved cultivar | Hap2 |
| 87 | Heihe 25 | Improved cultivar | Hap3 |
| 88 | Heihe 31 | Improved cultivar | Hap3 |
| 89 | Beifeng 9 | Improved cultivar | Hap3 |
| 90 | Hefeng 35 | Improved cultivar | Hap3 |
| 91 | Jiyu 39 | Improved cultivar | Hap3 |
| 92 | Zhangbaodadou | Landrace | Hap4 |
| 93 | Changmidou 30 | Landrace | Hap4 |
| 94 | Jiang Nong 416 | Improved cultivar | Hap4 |
| 95 | Kangxianchong 11 | Landrace | Hap5 |
| 96 | Heihe 14 | Improved cultivar | Hap5 |
| 97 | Gongye 04-L15 | Landrace | Hap6 |
| 98 | Huangjinyuan | Landrace | Hap6 |
| 99 | Dongnong 56 | Improved cultivar | Hap7 |
| 100 | Dongnong 59 | Improved cultivar | Hap8 |
